# Supplementary material for: Are carbon emissions trading and green financial instruments synergistic? -Comprehensive quantitative research based on content analysis
Source: PLoS One. 2024 Mar 7;19(3):e0298601. doi: 10.1371/journal.pone.0298601 (PMC10919601; doi:10.1371/journal.pone.0298601)
Supplement: S1 Data — (ZIP) [file pone.0298601.s001.zip › Data/地级市Z_F工作报告.zip!/║■─╧/║Γ╤⌠╩╨/└┤╫╘╣1⁄2╓┌║┼í╛┬φ┐╦╩2╛▌═°í┐_2018.docx]

各位代表：

现在，我代表市人民政府，向大会作政府工作报告，请予审议，并请各位政协委员和其他列席人员提出意见。

一、2017年工作回顾

2017年，面对经济下行压力和特大洪灾双重考验，市政府高举习近平新时代中国特色社会主义思想伟大旗帜，紧紧围绕迎接学习宣传贯彻党的十九大这条主线，在省委、省政府及市委的坚强领导下，在市人大、市政协的监督支持下，团结和带领全市人民，坚持稳中求进工作总基调，攻坚克难，砥砺前行，实现了新一届政府的良好开局。

——巩固了经济平稳增长的好局面。地区生产总值突破3000亿元，达3193亿元(预计数，下同)，同比增长8.5%；完成一般公共预算收入252.01亿元，同口径增长8.2%；规模工业增加值增长6.8%；固定资产投资增长14.3%；社会消费品零售总额增长11%；经济发展保持稳中有进、稳中向好的良好态势。财政总收入税占比、地方收入税占比分别达71.6%、55.9%，分别提高13.6个、12.9个百分点，108家入库税收过1000万元的企业实现税收翻番；推进高质量发展迈出坚实步伐。

——营造了积极干事创业的好氛围。纵深推进全面从严治党，健全容错纠错机制、推进正向激励、强化跟踪问效，综合施策治懒政、防怠政，提振了党员干部精气神，打响了政治生态“翻身仗”。广大党员干部尤其是领导干部积极主动作为、狠抓推进落实，干事创业的信心与勇气倍增，凝聚力、向心力和战斗力不断增强。

——引进了一批支撑发展的好项目。成功获批“中国制造2025”试点示范城市，扎实推进振兴实体经济“3311”工程计划、工业原地倍增计划。白沙绿岛军民融合产业园、水口山铜铅锌产业基地、比亚迪(衡阳)轨道交通智能制造产业园等一批支撑长远发展的战略项目次第落户，军民融合、有色金属、先进制造三大千亿级产业集群渐成雏形。

——树牢了勇于创新开放自信的好形象。被国务院明确为湘南地区中心城市；文明城市创建扎实推进，年度测评成绩大幅提升，成功创建国家园林城市；衡阳国家农业科技园区通过验收；成功举办2017衡州经济发展论坛，成立经济社会创新发展智库；衡阳的知名度、美誉度大幅提升。南岳机场航线达14条，旅客吞吐量突破47万人次，为建设湘中南地区航空枢纽奠定了坚实基础。着力构建亲清新型政商关系，严厉打击“官痞”“地痞”，助商、安商、富商的发展环境明显优化。

——办成了一批惠及广大人民群众的好实事。财政民生支出占一般公共预算支出比重达71.6%。11项15件重点民生实事任务圆满完成；脱贫攻坚成效显著，170个贫困村出列，9万多贫困人口脱贫，祁东县即将脱贫摘帽；城市黑臭水体得到有效整治；城市“六乱”大排查、大清理、大整改活动扎实开展，拆除“两违”建筑23.6万平方米；中心城区社区“三清三建”工作全面完成，新一轮提质升级工作扎实推进；市107国道绕城公路桥梁车辆通行费停止征收；改革发展成果惠及更多群众。

——夺取了抗洪救灾的全面胜利。以大无畏的气概迎战特大洪涝灾害，科学研判，精准施策，干群同心，军民同力，妥善转移安置群众5.62万余人，救助受灾困难群众3.9万人次。在湘江干流衡阳段全线超保证水位的情况下，实现了不垮一库、不溃一堤的防汛目标。

一年来，我们主要做了以下工作：

(一)突出植优补短，发展韧性明显增强。县域经济实力不断增强。七县市固定资产投资占全市比重达63.3%，提高1.2个百分点；衡南荣获“中国物流之乡”称号，祁东获评国家出口食品农产品质量安全示范区，常宁入选国家农产品质量安全县。农业品牌建设成效明显，“十百千万”现代农业发展工程大力推进，新增农产品“三品一标”75个。油茶产业快速发展，新造、新垦复油茶林面积分别达9.3万亩、19.87万亩，大三湘、中联、神农等企业不断壮大。工业经济活力不断迸发。重点产业发展壮大，六大重点产业完成工业总产值1247亿元，增长20%。工业投资活力增强，成功设立产业投资基金，高新南粤基金资产管理规模达37亿元，工业投资和工业技改投资分别增长16.1%、26%。绿色制造体系建设加快推进，单位工业增加值能耗下降4.5%。园区建设发展提速提质，基础设施建设累计投入91.76亿元，新增标准厂房96.88万平方米。旅游潜力不断发掘。全域旅游迈出坚实步伐，梦东方、汇景雁湖生态文旅小镇、天子山火文化公园、东洲岛、云鹫峰等重点项目加速推进，丽波国际酒店建成营业。新增3A级景区3个。岐山森林公园获评国家森林公园，南岳区获评国家重点生态功能区、国家级旅游业改革创新先行区。“厕所革命”三年行动计划共改扩建旅游厕所298座。全省首家工业主题博物馆建成开馆，工业旅游实现历史突破。乡村休闲旅游亮点纷呈，茶山坳镇入选全国美丽宜居小镇、全国特色小镇，曲兰镇获评全国经典文化小镇，雨母山景区获评全国人文生态旅游基地。接待国内外游客4800万人次，实现旅游总收入340亿元，分别增长15%、24%。

(二)突出改革开放，新生动能加快培育。供给侧结构性改革全面推进。去产能、去库存、去杠杆、降成本成效明显，“地条钢”生产企业全部关停，退出煤炭落后产能51万吨，处置完成国有僵尸企业5家；市属国有公司改革深入推进，债务风险整体可控；减税降费政策全面落实，累计为企业减负35亿元以上。农业供给侧结构性改革扎实推进，农村土地确权率达97.2%，耕地流转率居全省第一；粮食总产量稳定在330万吨以上；新型农业生产经营体系不断完善，新认定市级以上农业产业化龙头企业298家。关键领域改革步伐坚定。行政执法三项制度改革试点工作有序开展，耒阳综合行政执法体制改革经验在全省推介。不动产统一登记改革加快推进，地下车位发证进展顺利。国库集中支付电子化管理改革不断深化，预决算公开全面推行。公立医院综合改革深入推进，分级诊疗制度逐步建立。国资国企改革持续深化，“三供一业”分离移交有序推进。金融服务实体经济有力有效，市金融信用信息服务中心注册企业近6000家，撮合贷款逾130亿元；农信社银行化改革全面完成，村镇银行基本实现县域全覆盖。信用信息共享交换平台顺利建成。12家企业进入省重点上市后备资源库。商事、城管执法、生态文明等领域改革稳步推进。创新创业活力不断激发。大众创业、万众创新深入开展，新增创业主体2.7万个，市场主体达24.2万户。创建国家级博士后工作站2个、国家重点实验室1家，院士专家工作站达13家，专利申请量和授权量连续5年增长20%以上。湘南首个科技成果交易平台“火凤凰”成功开启，省级油茶产业技术创新战略联盟顺利落户，三叶中小企业创业基地获批国家小型微型企业创业创新示范基地，衡东获批国家返乡创业试点县。新业态、新模式不断涌现，新增高新技术企业51家，高新技术产业增加值增长16.8%。市留学生创业园和启迪之星企业孵化器入驻创新型企业217家。电子商务蓬勃发展，年交易额达130亿元。开放型经济水平显著提升。综合保税区稳健运行，衡阳跨境电商产业园投入运营，新开辟北粮南运通道2条。招商选资成效明显，实际利用外资12.5亿美元、内资380亿元。对外贸易实现历史性突破，40家企业外贸业绩破零，21家外贸企业实现业绩倍增，外贸进出口总额达283亿元，加工贸易总额居全省第一，富士康进出口总额居全省单体企业第一；对外经济合作遍及全球140多个国家和地区。

(三)突出城乡统筹，区域发展更加协调。区位优势日益凸显。西南云大城镇群纳入省城镇群产城融合综合试点。区域性物流贸易中心加快建设，衡缘、铁路口岸、白沙洲等物流园建设稳步推进。大浦通用机场建设进展顺利。怀邵衡铁路、瓦松铁路专用线、湘江二级航道二期工程、二环东路、东三环等项目加快建设，对外大畅通、对内大循环的综合交通运输体系更加完善。城市品位不断提升。新一轮城市总体规划、土地总体规划修编全面启动。滨江新区、来雁新城、陆家新区等新区建设有序推进。老城区雨污分流、道路提质改造项目全面完成，城区路网及配套设施不断完善。城市建成区实现旱厕清零。市餐厨废弃物资源化利用和无害化处理中心建成投产，县级以上城镇生活污水处理率达92.8%，城市生活垃圾处理率达100%。“三江六岸”生态绿地建设扎实推进，城市建成区绿化覆盖率达40.99%。城市管理更加精细。电动车上牌管理工作全面启动，交通秩序整治有力有效。中心城区社区“两化两体系”建设基本完成。城市停车设施不断完善。社区网格化管理工作机制进一步健全。餐饮油烟、垃圾焚烧等管理不断加强。县城管理提质升级，衡阳县获评国家卫生县城。城乡环境大幅改善。湘江保护和治理第二个“三年行动计划”扎实推进，河长制稳步实施，重点流域水污染治理成效明显，24个考核断面水质年均值达到Ⅱ类标准，县级以上集中式饮用水水源地水质达标率达100%。重点乡镇污水处理厂建设扎实推进。蓝天保卫战取得阶段性成效，城区空气质量优良率达79.2%。土壤污染状况详查全面完成，污染场地修复稳步实施。农村环境综合整治实现全覆盖，37个美丽乡村示范村建设、580个村农村环境综合整治任务全面完成。礼拜五秘书网

(四)突出坚守底线，民生福祉大幅提升。人民生活水平持续改善。城乡居民人均可支配收入分别增长8.5%、7.9%。开工建设城市棚户区23739套，改造农村危房8565户。提质改造农村公路976.42公里，建制村客运班线通达率达98.7%。解决12.21万贫困人口和27.98万非贫困人口的饮水难问题。新建成农村综合服务平台238个。社会保障体系不断健全。新增城镇就业5.94万人，新增农村劳动力转移就业4.6万人，城镇登记失业率控制在4.1%左右。五大险种参保基本实现全覆盖，企业退休人员养老金实现“十三连调”。机关事业单位退休人员养老金实现社会化发放。农村低保标准和扶贫标准实现“两线合一”，城镇居民医保与农村合作医疗合并工作如期完成，全民参保登记、社会保障兜底脱贫等工作经验在全省推广。各项社会事业协调发展。353所标准化学校创建任务圆满完成，市八中、环城南路小学获评首届全国文明校园，“全面改薄”惠及52.97万名学生，成功承办全国听障教育年会。基层医疗卫生服务体系不断健全，居民电子健康档案建档率达90.6%，计划生育工作稳步推进。公共文化服务体系不断完善，新闻出版、广播影视、文学艺术繁荣发展，耒阳、衡阳县荣获“中华诗词之乡”荣誉称号。成功承办2017年全国蹦床锦标赛暨第十三届全运会预选赛，实现我市承办国家A级赛事“零”的突破；圆满举办第十届中韩体育文化交流活动。工会、共青团、妇联、残联、气象、水文、移民、农开、民族、宗教、外事、侨务、港澳台、海关、检验检疫、文化市场综合执法、质监、畜牧水产、供销、地震等各项工作协调共进。底线工作基础全面夯实。环境保护方面，中央环保督察交办的512.5件信访件全部办结；生态保护红线划定工作顺利完成，南岳衡山国家级自然保护区内17家采矿企业全部停产关闭；永久基本农田保护率提升33个百分点；创新开展“四级联动”义务植树，完成造林30.56万亩，退耕还林还湿1640.34亩，全市森林覆盖率达47.6%。安全生产方面，“一单四制”制度有效落实，重点行业领域安全生产工作全面加强，森林防火成效明显，安全形势稳定向好。社会稳定方面，“平安行动”“畅安行动”“禁毒风暴”等工作扎实有效，十九大安保维稳任务圆满完成，南岳安保模式被公安部推介；中央和省交办的57件及市本级406件信访积案全面清零；防范和处置非法集资工作扎实开展，金融安全区创建稳步推进，守住了不发生区域性金融风险的底线。

(五)突出自身建设，政府形象不断优化。法治政府建设深入推进。第二部地方性法规《衡阳市城市市容和环境卫生管理条例》即将施行。政府合同管理、法律顾问、行政决策合法性审查等制度不断健全，依法行政水平明显提高。普法工作深入开展。行政执法指导案例制度基本建立。市县两级公共法律服务中心全面建成。依法完成2761个村(居)委会换届选举。珠晖区获评全国法治先进区。自觉接受人大及其常委会的法律监督和工作监督、政协的民主监督以及社会各界的监督，充分采纳各民主党派、工商联和无党派人士意见、建议，办理市人大代表议案1件、建议405件，办理市政协委员提案564件。行政效能持续提升。“两集中、两整合、两公开”改革深入推进，审批环节精简率达63.5%。“互联网+政务服务”正式启动，行政许可标准化全面推行，项目建设全程代办服务进一步优化。“数字衡阳”稳步推进，“12345”政府服务热线综合排名进入全国前30强。廉政建设不断加强。市政府系统实现派驻纪检机构全覆盖，“两学一做”学习教育常态化制度化成果不断巩固，党员干部作风持续改进。机构编制得到严控严管。财政监管、审计监督不断强化，风险防控、财政管理水平明显提高，市属国有企业资本运营、国有土地出让、招投标等领域监管全面加强。

国防动员和后备力量建设质量提高。优抚安置和民兵预备役建设得到加强，人防融合建设“衡阳模式”在全国推广，国防动员年度任务全面完成，军队有偿服务全面停止，军民融合发展呈现新局面。

各位代表!2017年成绩的取得来之不易，这是省委、省政府及市委正确领导的结果，是全市人民坚韧不拔、克难奋进的结果，是社会各界人士关心支持、大力帮助的结果。在此，我代表市人民政府，向全市广大工人、农民、知识分子、干部职工，向各民主党派、各人民团体，向驻衡部队、武警官兵和政法干警，向国内外所有关心支持衡阳发展的各界人士，表示诚挚的感谢和崇高的敬意!

在肯定成绩的同时，我们更要清醒地看到问题和不足：一是经济发展质量不优、效益不高、不平衡不充分等问题依然突出，实体经济面临不少困难，植优补短工作任重道远，制约经济社会发展的体制性、结构性矛盾并未根本解决。二是财政收支平衡难度加大，县域财政增收后劲不足，降低非税收入比重压力不小，脱贫攻坚、就学、就医、社会保障等民生领域还有大量工作要做。三是政府自身建设有待加强，办事效率不高、投资环境不优的状况仍未根本改变，部分公职人员不愿为、不敢为、不善为甚至乱作为现象还不同程度存在。对此，我们一定高度重视、认真研究、大力改进。

二、2018年主要任务

2018年是贯彻党的十九大精神的开局之年，是改革开放40周年，是全面建成小康社会、打赢脱贫攻坚战的关键一年，也是产业项目建设年。做好2018年政府各项工作意义重大、影响深远，必须深化认识、把握重点、加压奋进。

2018年政府工作的总体要求是：全面贯彻党的十九大精神，以习近平新时代中国特色社会主义思想为指导，认真落实中央、省委、市委决策部署，坚持稳中求进工作总基调，坚持新发展理念，紧扣社会主要矛盾变化，按照高质量发展的要求，以推进供给侧结构性改革为主线，深入实施创新引领开放崛起战略，统筹做好稳增长、促改革、调结构、惠民生、防风险各项工作，坚持一张好的蓝图干到底，着力打造现代产业强市、文化旅游名城、幸福和谐家园，推动经济社会持续健康发展，加快建设“五个新衡阳”、决胜全面建成小康社会。

2018年全市经济社会发展主要预期目标是：地区生产总值增长8%以上、争取达到8.5%，一般公共预算收入增长5%左右，固定资产投资增长14%左右，社会消费品零售总额增长11%左右，进出口总额增长20%左右，规模工业增加值增长7.5%左右，全体居民人均可支配收入增长8.5%左右，全面完成省里下达的节能减排、安全生产等约束性指标任务。

围绕以上目标，要重点做好以下工作：

(一)加快建设制造强市，着力振兴实体经济

全面落实振兴实体经济“3311”工程计划。加快白沙绿岛军民融合产业园建设，打造产城融合工业新城。抓好水口山铜铅锌产业基地建设，重点发展壮大铜铅锌、有色循环、铜压延等产业。抓好比亚迪(衡阳)轨道交通智能制造产业园建设，统筹推进盐卤化工及精细化工、新材料、新能源产业发展。充实上市企业后备资源库，支持南岳电控、恒飞电缆、恒生制药、金则利等优势企业上市。全力推动衡山科学城新技术、新产业、新平台、新业态发展，引导南华大学与衡山科学城共建大学科技园，打造全国一流的科技研发创新和成果交易中心。完善综合保税区大通道、大通关、大平台建设，培育服务贸易新业态，力争纳入湖南省自贸区申报范围。

着力发展先进制造业。加快“中国制造2025”试点示范城市建设，推进衡阳由工业大市向工业强市跨越。围绕先进制造产业建链补链强链，加快建滔电子信息产业园、富士康精密模具产业园和亚马逊中国制造基地等重点项目建设，打造优势产业集群。实施智能制造工程，依托特变电工、华菱衡钢、湖南机油泵、湖南天雁、南岳生物等重点企业开展智能制造示范，建设一批智能工厂。全面推进工业强基工程，着力破解我市制造业关键基础材料、核心基础零部件、先进基础工艺和产业技术基础瓶颈。优化工业经济结构，着力发展新一代信息技术、新材料、生物医药及高性能医疗器械、轨道交通、精密模具和机器人等新兴产业。全面实施绿色制造工程，引导传统产业加大技改投资，构建绿色制造体系。

做大做强工业园区。集中力量推进市高新区、白沙洲工业园、松木经开区提质发展。引导市直园区产业链条向县域延伸、配套企业落户县域园区，有序将部分重大项目布局到蒸湘南路、船山西路、衡祁干线两侧，实现县域园区和市直园区共建互补，加快产城融合步伐。大力推进园区体制改革，理顺园区体制机制，推动管理重心下移，扩大园区经济管理权限；创新推行灵活的用人制度、薪酬制度和激励制约机制。加快推进园区“多规合一”，着力促进总规、城规、土规、林规和园区产业发展规划统筹协调。

(二)全面深化改革开放，积极培育发展新动能

深化供给侧结构性改革。大力破除无效供给，积极稳妥、依法依规处置“僵尸企业”，持续化解过剩产能；巩固“地条钢”生产企业、落后小煤矿、烟花爆竹行业等关停退出成果，防止死灰复燃。坚持房子是用来住的、不是用来炒的定位，加快建立多主体供应、多渠道保障、租购并举的住房制度；鼓励农业转移人口等新市民到市区和县城购房租房；有序解决房地产历史遗留问题；促进房地产市场平稳健康发展。深化要素市场化配置改革，进一步打好减税、降费、降低要素成本等“组合拳”，继续清理涉企收费，降低企业用能及物流成本，促进实体经济降本增效。深化农业供给侧结构性改革，扎实推进林权和农村不动产登记工作，抓好常宁全国农村集体产权制度整县推进改革试点；加快农村集体产权制度改革，全面完成农村土地确权登记工作；深入推进粮食收储制度改革；壮大新型农业经营主体，健全农业社会化服务体系。

加快关键领域改革。深化金融体制改革，积极发展股权融资、私募基金、企业债券等直接融资；加快组建市金融控股公司，不断完善市金融信用信息服务中心功能，提升金融服务实体经济的能力。深化投融资体制改革，健全投融资公司绩效考核制度，鼓励投融资公司支持产业发展；在污水处理、垃圾收集、城市地下综合管廊、黑臭水体整治、旅游交通等基础设施建设领域大力推行PPP模式。完成驻衡中央、省属企业“三供一业”分离移交，推动国有企业完善现代企业制度，做强做优做大国有资本。深化商事制度改革，放宽服务业准入限制，完善市场监管体制。探索建立城建领域重大项目联席会议制度。全市域推进综合行政执法体制改革。深化医药卫生体制改革，抓好公立医院改革。完成环保监测监察执法机构垂直管理体制改革。完善统计体制，增强统计服务功能，做好第四次经济普查工作。加快户籍制度改革。进一步理顺市区两级管理体制，赋予城区更大自主权。深化财政评审体制改革，探索建立重点项目联合评审制度。完善征拆奖惩机制。统筹推进省直管县、经济发达镇行政管理、审计管理、防灾减灾、生态文明等体制改革。

加快建设国家创新型城市。实施科技强市战略。重点围绕高端装备制造、新材料、核产业、生物医药等领域开展核心关键技术攻关，培育一批具有创新能力的排头兵企业，提高产业核心竞争力。抓实高新企业培育认定。支持市高新区围绕创建国家创新型特色园区，打造总部经济区、现代产业核心区、国家级产城融合示范区。支持校地、校企共建工程技术研究中心、重点实验室、院士专家工作站等研发平台；支持蒸湘区双创园区易创空间发展，鼓励市留学生创业园、启迪之星企业孵化器等申报国家级众创空间、孵化器。创新人才工作体制机制，实施创新人才集聚工程，加大财政对人才发展的投入，引进和培养一批科技创新领军人才。加强知识产权保护，提高科技成果转换和利用水平。

着力打造内陆开放高地。全面落实《衡阳市促进招商引资工作的若干措施》，对接世界500强、中国500强和民营500强企业，深入开展“引老乡、回故乡、建设衡阳新家乡”活动，引进一批带动支撑衡阳长远发展的产业项目。大力发展加工贸易。着力培育外贸企业，扶植一批有自主知识产权、自主出口品牌的大型外贸集团和中小型外贸企业，优化外贸产品结构，提高出口产品的附加值和竞争力。鼓励企业“走出去”，支持特变电工、五矿铜业、华菱衡钢、瑞达电源等企业在境外设立营销网络，参与国际产能合作。放大临铁、临港、临空优势，高效运行“五定班列”“无水港”等开放平台；力争南岳机场新增航线15条以上，申报建设航空口岸，打造湘中南地区航空枢纽。积极申办国内外大型会议、论坛、会展，发展同发达国家或先进地区友好城市关系。

(三)落实乡村振兴战略，推动区域协调发展

发展现代农业。落实粮食安全行政首长责任制，实施优质粮食工程。加快推进农产品加工业发展，力争农副产品加工业总产值增长15%以上。启动全国性农产品交易展示中心建设。支持发展“一县一业”“一乡一品”，逐产业打造1-2个拳头品牌。大力建设油茶、茶叶、黄花菜等优质农副产品主产区和供应基地，支持大三湘、中联、神农等企业强化精深加工，培育衡阳油茶全国知名农产品区域公用品牌，打造全国油茶第一强市；提升“南岳(衡山)云雾茶”“祁东黄花菜”等区域公用品牌价值。支持角山米业、金雁米业、绿海粮油等龙头企业做大做强，力争新增农产品“三品”50个以上、地理标志产品2个以上。发展“农业+休闲旅游”“农业+电商”等新业态，促进一二三产业融合发展。坚持质量兴农、绿色兴农，狠抓农产品质量安全，推进农产品质量安全示范县市建设。加强中低产田改造、高标准农田建设，推进沟渠塘坝清淤，抓好重点水利基础设施建设。提升农业机械化和农业装备水平，加大农业科技应用推广及新型职业农民培育力度，完善农业保险制度，建立健全农业防灾减灾体系，不断夯实现代农业发展基础。

做强县域经济。坚持宜农则农、宜工则工、宜游则游，支持各县市分别打造1-2个具有鲜明特色的主导产业。借鉴先进地区发展经验，科学制定园区发展规划，完善配套设施及管理服务，引导项目向县域园区集中，打造一批产城融合、产业链完整的现代产业园区。加快西渡高新区、界牌陶瓷工业园、归阳工业园、耒阳经开区等县域园区建设。实施县城建设攻坚行动，加快县城扩容提质，细化县城功能分区，完善城镇水、电、路、讯等基础设施和环卫、绿化等配套设施。推进全省产城融合城镇群综合试点。支持各县市抓好1-2个特色集镇建设，打造一批工业强镇、农业重镇、商贸旺镇、旅游名镇，增强对农业转移人口的吸引力。

加大美丽乡村建设力度。科学制定乡村振兴战略规划。加快茶山坳、雨母山、横江、萱洲等美丽乡村示范片建设。有序推进田园综合体建设，打造蒸湘南路、船山西路美丽乡村示范廊道，完成省下达的37个美丽乡村示范村、340个人居环境示范村创建任务。加大农村危房改造、“空心房”整治、农村电网改造升级、农村道路提质改造、乡村亮化等工作力度。加强乡镇饮用水水源保护。深入推进农村环境综合整治，扎实开展秸秆禁烧和垃圾清运专项行动，打好农村面源污染防治攻坚战。推进农村“厕所革命”，全面整治农村村容村貌。严格村民建房管理。强化村庄规划编制，实现城区、县城周边的村发展规划全覆盖。探索农村集体经济发展新途径，增强村集体组织服务功能。健全自治、法治、德治相结合的乡村治理体系。

坚决打好脱贫攻坚战。巩固前期脱贫成果，保证现行标准下的脱贫质量，瞄准特定贫困群众精准帮扶，向深度贫困地区聚焦发力。落实“六个精准”要求，力争108个村出列、7万人以上脱贫、10824人易地扶贫搬迁、贫困村居民人均可支配收入增长10%以上。注重扶贫和扶志、扶智、扶技、扶艺相结合，激发贫困人口内生动力。深入实施就业扶贫工程，增加农村公路养护、乡村卫生保洁、护林员等公益性岗位，拓展困难群众就业渠道。落实十大扶贫举措，按照“四跟四走”思路，强化油茶种植、畜禽养殖等产业扶贫。扎实推进农村“四类对象”危房改造。确保符合政策规定的贫困人口全部纳入农村低保范围。对已脱贫对象开展“回头看、回头查、回头帮”，实行攻坚期内脱贫不脱政策。实施贫困村基础设施建设三年行动计划，加强脱贫攻坚自然村通水泥(沥青)路建设，确保所有贫困村全部通电、通路、通电视、通宽带。

(四)积极对接市场需求，深度挖掘消费潜力

加速发展全域旅游。加快南岳衡山旅游经济圈建设，力争南岳区成功创建国家全域旅游示范区，擦亮以南岳为龙头、多个旅游综合体与美丽乡村生活体验相匹配的全域旅游品牌。深化“引客入衡”工程，全年接待国内外游客、旅游总收入分别增长15%、24%以上。挖掘衡阳旅游资源和文化底蕴，抓好石鼓书院提质改造，加快梦东方、东洲岛、草桥文化风情街、汇景雁湖生态文旅小镇等项目建设，推进红色旅游教育基地建设，新创4A级景区1个以上、3A级景区2个以上。支持各县市对接南岳打造精品旅游景区和线路，鼓励有条件的县市区举办旅游节会活动。深入推进“互联网+旅游”，大力发展美丽乡村生活体验游，促进线上推广与线下管理融合发展。引入顶级旅游策划公司，强化全域旅游营销推广，加大在央视等国内一流媒体的旅游推介力度。完善“吃住行游购娱康养学”旅游产业链条，推介“衡东土菜”“南岳素菜”等特色产品。深化旅游“厕所革命”，完善道路、游客服务中心等配套设施。规范旅游市场，整治景区秩序，提升旅游服务。

推进服务业发展壮大。做强现代物流业，加快唯品会、衡缘、铁路口岸、白沙洲、圆通速递湘南总部基地等物流园建设，大力引进和培育第三方物流企业，支持品牌快递企业在衡建立区域转运中心。围绕高端装备制造、汽车零部件、生物医药、盐卤化工以及钢管材加工等领域，培育研发设计服务产业。积极发展多层次金融产业，优化金融生态环境，吸引各类金融机构来衡设立分支机构，打造金融集聚区，提升金融供给质量。着力推进电子商务、健康养老、全民健身等现代服务业发展，提升供给质量。加快建设衡阳国际会展中心、衡阳国际吉利汽车文化城，着力发展总部经济、汽车后市场等新兴业态。

推进消费提质升级。围绕华耀城、崇盛国际、万达广场、连卡福等城市综合体，积极培育消费新热点。积极引导企业增品种、提品质、创品牌，培育具有鲜明特色、较高品质、较强竞争力的“雁字号”产品。大力推进品质消费、个性化消费、服务消费、信用消费、信息消费，支持和引导共享经济健康快速有序发展。积极拓宽农村消费市场，打通城乡供需渠道，以农村电商为发展基础，推动农产品线上营销与线下流通融合发展。着力改善消费环境，保护消费者权益，让老百姓便捷消费、放心消费。

(五)做大做优中心城区，努力打造省域副中心城市

拓展城市发展空间。抓好新一轮城市总体规划修编，推进中心城区区划调整。加大来雁新城、滨江新区、酃湖高铁新城、陆家新区、南湖生态新城等新区开发力度。完善蒸水以北片区基础设施。高位推动衡山科学城、梦东方、雨母山片区融合发展。推进107国道改造，全面建成二环路，开工建设北三环；加快湘江二级航道二期工程、大浦通用机场、祁东归阳千吨级码头建设，启动湘江衡阳至永州三级航道改扩建，扎实推进湘桂线衡阳段改线工程前期工作，力争怀邵衡铁路衡阳段主线、瓦松铁路专用线建成通车；加快衡阳-祁东等快速干线、常祁高速公路及国省干线公路建设。

提升城市建管水平。坚持精美建设、精致管理，扎实开展“城市双修”工作，建设海绵城市、管廊城市、智慧城市，构建城市防洪防涝、山洪地质灾害防御体系。基本完成中心城区主次干道提质改造；打通城市断头路，科学设置单行线，逐步实现城市道路“微循环”；加快城市停车设施及智能停车平台建设，着力解决城市“停车难”问题。提质建设城市供水及能源通道。加强城市二次供水设施建设及管理，确保城市供水安全。加快城区污水治理，全面封堵湘江、蒸水城区段排污口，完成角山、松木、酃湖污水处理厂建设及城市黑臭水体治理任务。加大旧城改造力度，合理处置D级危房，扎实推进农贸市场标准化改造，积极打造中心城区社区“三清三建”升级版。加大重大违法建设处置力度，完成城市建成区违法建设“五年清零”行动目标任务。抓好高铁衡阳东站、衡阳火车站和学校、医院周边环境整治。持续推进殡葬改革，巩固和扩大中心城区烟花爆竹限放成果。加快城市生活垃圾焚烧发电厂二期、城乡生活垃圾收运体系、动物园异地搬迁等项目建设。推进“三江六岸”滨水区域规划建设管理，加快雁栖湖公园、酃湖公园建设，提质改造南郊公园。

提高城市文明程度。锲而不舍创建全国文明城市，抓好国家卫生城市、全国交通管理模范城市、国家环境保护模范城市、全国社会治安综合治理先进城市、全国双拥模范城市等单项城市荣誉品牌的创建。大力宣传党的十九大精神和社会主义核心价值观，广泛宣传各级各类道德模范“身边雷锋·衡阳好人”的典型事迹，弘扬正能量，传播真善美。加强社会公德、职业道德、家庭美德、个人品德建设。倡导移风易俗、树立文明新风，开展星级文明社区、文明村镇、文明单位、文明校园、文明家庭评选表彰活动。坚持创建为民、创建惠民，开展丰富多彩的市民素质教育活动，引导广大市民树立文明观念、争当文明公民、展示文明形象。

(六)践行绿色发展理念，建设生态优美新衡阳

推进绿色循环低碳发展。深入推进节能减排，加快碳排放交易市场建设，创新排污权交易制度，抓好排污许可证核发工作。大力发展绿色环保产业，加快住宅产业化发展，建立健全绿色低碳循环发展经济体系。落实最严格的水资源管理制度，推进农业水价综合改革，打造节水型城市。推动国家级循环化改造示范园区建设，严格工业园区项目建设生态环境监管。规范工业固体废物特别是危险废物管理，提高工业固体废物利用率。调整运输结构，提升铁路货运、清洁能源货运能力。鼓励绿色出行，推进公共交通设施建设，加大新能源公交推广力度，完善充电桩等基础设施，加强公共自行车租赁系统管理。

着力打好环境治理战役。深入实施“水十条”，扎实推进湘江保护和治理第二个“三年行动计划”，启动实施“一江四水”净水行动，抓好退耕还林还湿。全面推行河长制，完成禁养区畜禽规模养殖场搬迁、关闭和适养区标准化改造，加快推进畜禽养殖废弃物处理和资源化利用；加强饮用水水源保护，确保13个县级以上饮用水水源地水质达标率稳定为100%，湘江干流考核断面水质年均值稳定在Ⅱ类标准以上。推行环境治理PPP模式，探索区域环境综合治理托管服务。加强集镇“五乱”治理，按照“一乡(镇)一策”全面推进乡镇污水处理设施建设。持续深化大气污染防治，全面淘汰城区10蒸吨及以下燃煤锅炉，加大建筑工地、道路扬尘和餐饮油烟整治力度，严格开展机动车环保检测，力争城区空气优良天数达300天以上。全面实施“土十条”，扎实推进土壤污染场地和耕地污染治理修复，加大对水松、大浦等重点地区重金属污染土壤的治理和修复力度。

加强生态建设和环境保护。牢固树立“绿水青山就是金山银山”发展理念，建立健全生态文明建设目标评价考核与环境联合执法工作机制。加快国家级生态文明先行示范区建设。加强自然保护区特别是南岳衡山国家级自然保护区监督管理，有序推进保护区范围和功能区划纠错调规。加快耒水、横江、洣水、幸福河等湿地公园建设。落实耕地保护制度，提升耕地质量。严守生态保护红线，严格执行主体功能区制度。开展第二次全市污染源普查工作。开征环境保护税。推进森林城市建设，实施生态功能优化行动，统筹山水林田湖草系统治理，启动大规模国土绿化行动，推进“裸露山地”造林绿化、矿区复绿等工作，抓好独立工矿区改造搬迁、采煤沉陷区综合治理，力争森林覆盖率达482%。

(七)传承弘扬优秀文化，着力打造历史文化名城

传承历史文明。发掘并弘扬火文化、书院文化、红色文化、抗战文化、大雁文化等优秀传统文化。启动历史文化街区整治工程，加大文化遗产、历史建筑的保护和修缮力度，完成来雁塔、珠晖塔、接龙塔、申公馆、彭玉麟墓庐、邺侯书院、三甲种工校等文物保护单位修缮工程，推动酃县故城、衡州窑、云集窑、张家山公园、水口山工人运动纪念馆等建设。完成历史文化名城保护规划编制工作。完成市博物馆新馆、湘南学联纪念馆展厅选址，发展非国有博物馆。实施传统戏曲和传统工艺振兴计划。启动纪念王船山诞辰400周年系列活动筹备工作。办好南岳庙会、界牌火灯节、泉湖二月八等优秀民俗文化节会活动，重点谋划南岳衡山民俗文化生态保护区建设。加强历史文化村镇和传统村落保护。

发展文化事业。完善公共文化服务体系，提高公共文化服务保障能力，促进基本公共文化服务标准化、均等化。深入实施文化惠民工程，加强基层文化服务中心建设，巩固“村村响”“户户通”“送戏送电影下乡”成果。创建第二批省级公共文化服务体系建设示范区。推进全民阅读，合理布局建设24小时自助图书馆，建设“书香雁城”。加强文化、新闻出版、广播影视等市场监管，深入开展“扫黄打非”，不断净化文化市场环境。加快市图书馆、市科技馆、市影剧艺术中心、市青少年活动中心等项目建设。

壮大文化产业。扩大“石鼓书院大讲坛”影响，争取开拍《衡阳保卫战》电影，不断推出优秀文艺作品。壮大新闻出版、广播影视等传统文化产业，发展新型文化业态。大力发展“文化+”，繁荣文化创意产业，加快天子山火文化公园、船山书院文旅开发、茶山坳运动休闲特色小镇、渣江玉麟文化古镇、新市古镇等建设。实施“一县一品”“一乡一韵”区域特色文化建设工程。

(八)提高保障和改善民生水平，增进民生福祉

优先发展教育事业。推动城乡义务教育一体化发展，高度重视农村义务教育，保障进城务工人员子女平等接受义务教育；基本消除超大班额，完成义务教育标准化学校和教学点建设任务。着力解决中小学生课外负担重等问题。制定并实施第三期学前教育行动计划，加快建设公办幼儿园，发展普惠性幼儿园。集中力量办好华新实验中学、市九中、市铁一中三所公办中学，加快市十七中迁建、市七中搬迁，支持各县市建设高质量公办中学，缓解“择校热”问题。逐步普及高中阶段教育。大力发展职业教育，推进市职业教育产教融合基地和市示范性综合实践基地建设，力争衡阳幼师、耒阳师范、市艺术学校合并升格为高等专科学校。支持在衡高校建设一流大学和一流学科，加快南华大学新校区建设。完成“全面改薄”任务。稳步发展特殊教育。支持和规范社会力量兴办教育。培养高素质教师队伍，倡导全社会尊师重教。建设学习型社会，提高市民素质。

积极稳就业促增收。提供全方位公共就业服务，加强劳动保障平台建设，多渠道推进高校毕业生、农村转移劳动力、退役军人等重点群体充分就业、自主创业。深入推进创业带动就业，加强创业孵化基地建设。全面落实更加积极的就业政策，加大援企稳岗力度，确保城镇登记失业率控制在4.5%以内。继续实施“雁归兴衡”行动计划。加大高技能人才队伍建设力度，树立“技能就业”的良好导向，年内培训技能人才1000名以上，培训贫困家庭“两后生”500人以上，吸引在衡大中专院校毕业生在衡阳就业发展、安家落户。

完善社会保障体系。继续抓好“五险统征”改革。推进企业社保制度改革，落实各项社保政策。建立市内异地就医结算平台。健全社会救助体系，提高城乡低保标准和月人均救助水平，落实重大疾病医疗救助和临时救助制度。发展社会福利和慈善事业，健全农村留守老人、妇女、儿童关爱保护体系。完善住房保障体系及棚户区改造工作机制，完成年度棚户区改造任务；加快公租房配套工程建设，确保需交付使用的公租房达到分配入住条件。

提高全民健康水平。推进健康衡阳建设，整合医疗资源力量，打造湘南区域性医疗中心和康养基地。加强基层医疗卫生服务体系建设。组建家庭医生团队，完善家庭医生签约服务。健全分级诊疗制度，延伸医联体内涵。积极应对人口老龄化，鼓励社会资金进入城乡养老领域，健全居家养老和社区养老服务网络。完善医疗纠纷调解机制，依法严惩涉医犯罪和“医闹”行为。落实全面两孩等国家生育政策，深化计划生育服务管理改革，加快市妇幼保健院新院建设，提升妇幼健康服务能力。广泛开展全民健身运动。积极发展竞技体育。高水平办好省十三运会和省第十届残运会。

着力防范金融和政府性债务风险。加强对薄弱环节和担保、小贷、民间中介等融资机构的全流程监管，坚决打击非法集资、金融诈骗等违法违规金融活动，积极化解民间融资风险、金融机构不良风险，不断优化金融生态环境，着力打造金融安全区，坚决守住不发生区域性金融风险的底线。摸清政府性债务尤其是隐形债务底子，严格控制增量，逐步消化存量。加强社会信用体系建设，健全守信激励、失信惩戒机制。

加强和创新社会治理。完成“雪亮工程”建设，完善社会治安立体化、现代化防控体系，深入推进“平安行动”“畅安行动”“禁毒风暴”“扫黑除恶”等专项行动；全面推行网格化服务管理、电动车登记管理，着力打造“平安衡阳”“法治衡阳”升级版。落实信访工作责任制，开展信访法治建设年活动，促进信访工作回归群众工作本源和法治轨道。加强食品药品监管。落实安全生产责任制、“三个责任清单”，抓好重点行业领域专项整治，坚决遏制较大以上生产安全事故。加强防灾救灾减灾能力建设，基本消除重大地质灾害隐患，完善应急管理体系，提高突发事件防控处置能力。依法管理民族宗教事务和场所，持续开展民族团结进步创建“六进”工作。统筹发展其他社会事业，确保社会大局和谐稳定。

强化国防和后备力量建设。推进军民融合深度发展，开展全民国防教育和“双拥”共建活动，认真落实军转安置任务，支持国防军队改革，扎实抓好国防动员、军事设施保护、民兵预备役、武警、人防等工作。

三、努力建设人民满意的服务型政府

打铁必须自身硬。进入新时代，开启新征程，必须坚定不移加强政府自身建设，打造务实、高效、诚信、廉洁的服务型政府。

(一)坚持不忘初心，增强科学发展本领。牢固树立“四个意识”，自觉把习近平新时代中国特色社会主义思想贯穿到改革发展稳定各个领域。始终牢记根本宗旨，大兴调查研究之风，全心全意为群众办实事、解难事，不断满足人民群众日益增长的美好生活需要。切实强化担当意识，提高应对各种复杂矛盾、风险隐患的能力，对改革发展中的各种挑战、人民群众关切的重大问题，敢于知难而进、迎难而上、克难而行，坚决把中央、省、市决策部署落实到行动上、体现在工作上、彰显于业绩上。

(二)树牢法治思维，增强依法行政本领。加强和改进政府立法工作。在政府系统普遍建立法律顾问、公职律师制度。完善法治政府建设考评体系。加大普法工作力度。加快公共法律服务体系建设，支持和帮助企业依法妥善处理各类纠纷。自觉接受人大及其常委会的法律监督、工作监督和政协的民主监督，认真办理人大代表议案、建议和政协委员提案。严守法定权限和程序，发挥决策咨询机构和智库的作用，推进依法决策、科学决策、民主决策。严格、规范、公正、文明执法，全面落实行政执法三项制度，依法维护人民群众合法权益。

(三)提高行政效能，增强狠抓落实本领。进一步加强行政效能监察和审计监督，健全重大决策部署执行情况督促检查和专项督查制度，着力提高政府执行力。科学调整绩效考核指标和办法，强化考核结果的运用。深化“放管服”改革，持续推进“两集中、两整合、两公开”改革，加强审批服务标准化建设。扎实开展“最多跑一次”改革，不断提高政府办事效率。依托全市电子政务云平台，全面推行“互联网+政务服务”，强力推进政府信息资源共享和数据利用，提升“12345”政府服务热线服务质量。清理、督办、整改招商引资违约失信问题。着力整治行政不作为、慢作为、乱作为行为。完善干部考核评价机制，健全容错纠错和正向激励机制，为干部大胆创新探索撑腰鼓劲。

(四)恪守廉政底线，增强拒腐防变本领。认真落实全面从严治党主体责任和“一岗双责”，深入推进政府系统党风廉政建设和反腐败斗争。积极稳妥推进监察体制改革。完善财政资金支出、工程项目招投标等领域制度建设，不断提升财政资金的使用效益和水平。严禁领导干部和国家公职人员参与涉矿涉砂涉房地产等非法入股、地下经营等活动。坚持以上率下，扎实开展“不忘初心、牢记使命”主题教育，驰而不息抓好作风建设，坚决防止“四风”问题反弹回潮。着力构建亲清新型政商关系，规范部门入园、入企检查视察行为，切实减轻企业负担；持续严厉打击“官痞”“地痞”，对妨碍司法公正、阻碍项目建设行为保持“零容忍”，为一切市场主体创造公开、公平、公正、可预期的发展环境。

各位代表!奋斗成就梦想，实干赢得未来。让我们紧密团结在以习近平同志为核心的党中央周围，在市委的坚强领导下，团结带领全市人民，以更加昂扬向上的精神状态，锐意进取，开拓创新，真抓实干，努力为加快建成全面小康衡阳而不懈奋斗!
